# Synergistic Effect of Hyaluronate Fragments in Retinaldehyde-Induced Skin Hyperplasia Which Is a Cd44-Dependent Phenomenon

**DOI:** 10.1371/journal.pone.0014372

**Published:** 2010-12-16

**Authors:** Laurent Barnes, Christian Tran, Olivier Sorg, Raymonde Hotz, Denise Grand, Pierre Carraux, Liliane Didierjean, Ivan Stamenkovic, Jean-Hilaire Saurat, Gürkan Kaya

**Affiliations:** 1 Department of Dermatology, University of Geneva, Geneva, Switzerland; 2 Institute of Pathology, University of Lausanne, Lausanne, Switzerland; Tufts University, United States of America

## Abstract

**Background:**

CD44 is a polymorphic proteoglycan and functions as the principal cell-surface receptor for hyaluronate (HA). Heparin-binding epidermal growth factor (HB-EGF) activation of keratinocyte erbB receptors has been proposed to mediate retinoid-induced epidermal hyperplasia. We have recently shown that intermediate size HA fragments (HAFi) reverse skin atrophy by a CD44-dependent mechanism.

**Methodology and Principal Findings:**

Treatment of primary mouse keratinocyte cultures with retinaldehyde (RAL) resulted in the most significant increase in keratinocyte proliferation when compared with other retinoids, retinoic acid, retinol or retinoyl palmitate. RAL and HAFi showed a more significant increase in keratinocyte proliferation than RAL or HAFi alone. No proliferation with RAL was observed in CD44^−/−^ keratinocytes. HA synthesis inhibitor, 4-methylumbelliferone inhibited the proliferative effect of RAL. HB-EGF, erbB1, and tissue inhibitor of MMP-3 blocking antibodies abrogated the RAL- or RAL- and HAFi-induced keratinocyte proliferation. Topical application of RAL or RAL and HAFi for 3 days caused a significant epidermal hyperplasia in the back skin of wild-type mice but not in CD44^−/−^ mice. Topical RAL and HAFi increased epidermal CD44 expression, and the epidermal and dermal HA. RAL induced the expression of active HB-EGF and erbB1. However, treatment with RAL and HAFi showed a more significant increase in pro-HB-EGF when compared to RAL or HAFi treatments alone. We then topically applied RAL and HAFi twice a day to the forearm skin of elderly dermatoporosis patients. After 1 month of treatment, we observed a significant clinical improvement.

**Conclusions and Significance:**

Our results indicate that (i) RAL-induced *in vitro* and *in vivo* keratinocyte proliferation is a CD44-dependent phenomenon and requires the presence of HA, HB-EGF, erbB1 and MMPs, (ii) RAL and HAFi show a synergy *in vitro* and *in vivo* in mouse skin, and (iii) the combination of RAL and HAFi seems to have an important therapeutic effect in dermatoporosis.

## Introduction

CD44 is a facultative cell surface proteoglycan expressed as several isoforms [1], and the principal cell surface receptor of hyaluronate [3], [4] (HA), the major component of the extracellular matrix [5]. In our previous study we have shown that CD44 is implicated in the regulation of keratinocyte proliferation and the local HA metabolism in mice [6].

We have recently shown that the epidermal hyperplasia induced by topical retinoids was accompanied by an increased expression of CD44 and hyaluronate synthases and associated with an increase in epidermal and dermal HA in mouse skin [7]. We have also shown that the decrease of the expression of CD44 and hyaluronate induced by UVA and UVB in mouse epidermis is counteracted by topical retinoids [8]. Topical application of one of these retinoids, retinaldehyde (RAL), a natural retinoid immediate precursor of retinoic acid (RA), restores the epidermal thickness and CD44 expression which are correlated with clinical improvement in lichen sclerosus et atrophicus (LSA) lesions [9], where the epidermal expression of CD44 has been shown to be decreased or absent [10].

RAL has been shown to exert biological activity in mouse and human skin [11], [12]. It has been shown that the epidermal hyperplasia induced by topical retinoids was linked to a RA receptor (RAR)-dependent heparin-binding epidermal growth factor (HB-EGF) paracrine loop [13]. It has also been shown that a heparan sulfate-bearing variant of CD44 (CD44v3) recruits proteolytically active matrix metalloproteinase 7 (MMP-7), the precursor of HB-EGF (pro-HB-EGF) and its receptor, erbB4 to form a complex on the cell surface [14]. We have recently shown that CD44 is colocalized with another HB-EGF receptor, erbB1 on keratinocytes [15]. We have also shown that topically applied HAF of intermediate size (HAFi) traverse the skin and induce a CD44-dependent biological effect characterized by a skin regeneration in mice and elderly human patients showing dermatoporosis, the holistic word for human skin fragility and an emerging clinical problem due to chronological aging, long-term sun exposure and chronic use of corticosteroids [15], [16], [17].

To see whether retinoid-induced epidermal hyperplasia via HB-EGF was a CD44-related phenomenon, we compared the effect of different retinoids on in vitro proliferation of keratinocytes DBA/1 mice. We further tested the effect of the blockade of HA synthesis, HB-EGF, erbB1 and MMPs including MMP-7 on the proliferation of the keratinocytes of SKH1 hairless, DBA/1 and CD44-deficient (CD44^−/−^) mice. We also analyzed the effect of RAL on the epidermal hyperplasia in SKH1 hairless, DBA/1 and CD44^−/−^ mice, and in vivo expression of CD44v3, MMP-7, HB-EGF and its receptors in SKH1 hairless mice. To address the possibility that RAL and HAFi may have a synergy on keratinocyte proliferation and epidermal hyperplasia, we examined the effect of the combination of HAFi and RAL on the mouse skin *in vitro* and *in vivo*, and on atrophic skin of elderly patients with dermatoporosis.

## Results

### RAL is the most potent retinoid to induce keratinocyte proliferation

**Figure 1:**
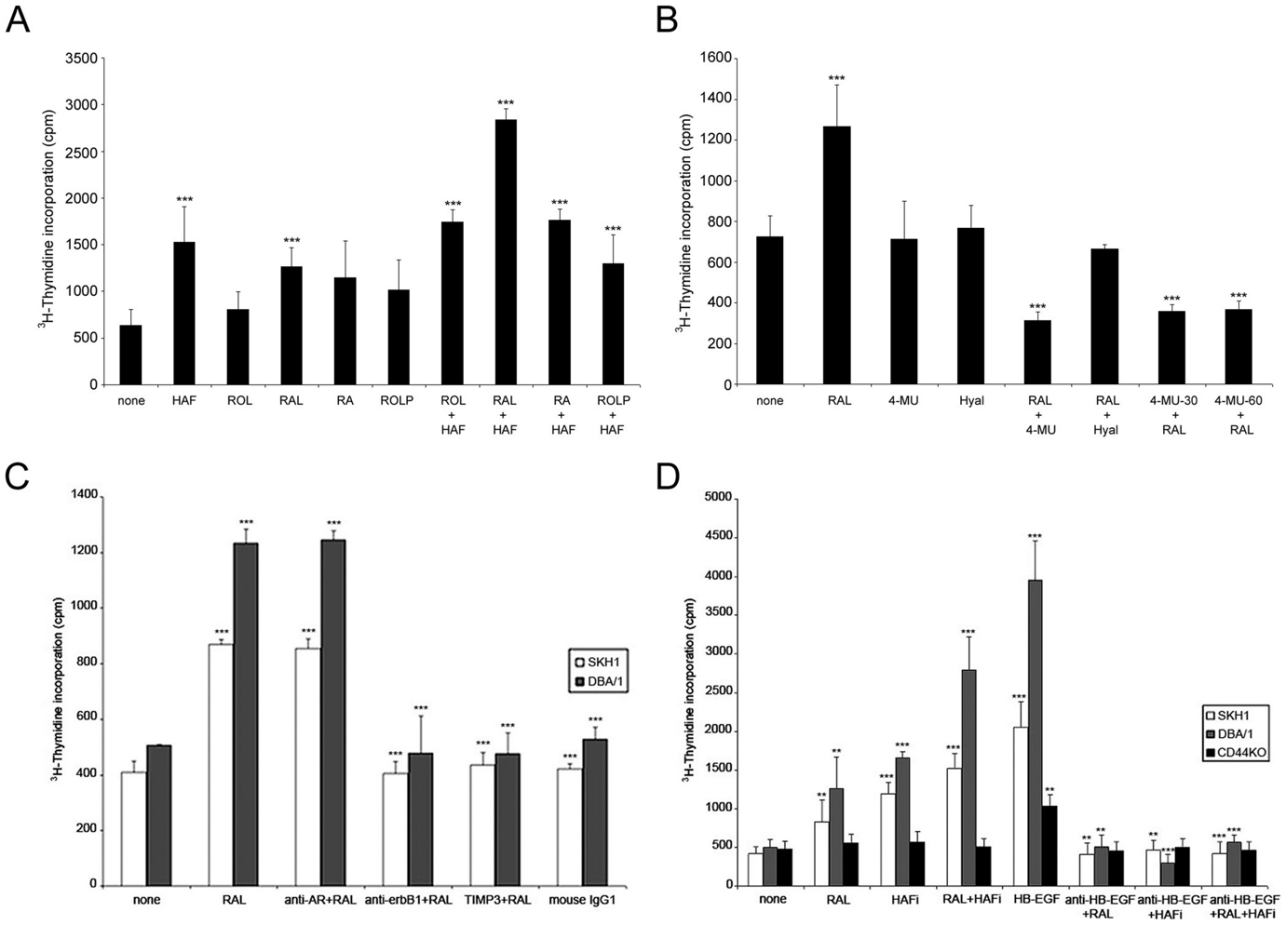
The effect of retinoids and HAFi *in vitro*. **A.** RAL is the most potent retinoid to induce keratinocyte proliferation. Keratinocytes from DBA/1 mice were cultured in 96-well plates. On day 2 of culture, HAFi (100 µg/ml), RAL (2 µM), RA (2 µM), ROL (2 µM), ROLP (2 µM), RAL+HAFi, RA+HAFi, ROL+HAFi or ROLP+HAFi was added to the cultures. 48 hrs later 1 µCi of [^3^H]thymidine was added to each well. All experiments were done in triplicate and repeated 5 times. The results are presented as the mean incorporated counts per minute ± SEM of three wells per group. ***p<0.001(HAFi versus none; RAL versus none, RA, ROL and ROLP; RAL+HAFi versus all; ROL+HAFi versus none; RA+HAFi versus none; ROLP+HAFi versus none) (student's t test). **B.** RAL-induced HA synthesis is required for keratinocyte proliferation. Keratinocytes from DBA/1 mice were cultured in 96-well plates. On day 2 of culture, RAL (2 µM), 4-MU (0.6 mM) [alone or together with RAL (RAL+4-MU) or 30 (4-MU-30+RAL) or 60 (4-MU-60+RAL) minutes before RAL] or hyaluronidase (1 U/ml) [alone or together with RAL (RAL+Hyal)] was added to the cultures. 48 hrs later 1 µCi of [^3^H]thymidine was added to each well. All experiments were done in triplicate and repeated 5 times. The results are presented as the mean incorporated counts per minute ± SEM of three wells per group. ***p<0.001(RAL versus none, RAL+4-MU, 4-MU-30+RAL and 4-MU-60+RAL; RAL+4-MU, 4-MU-30+RAL and 4-MU-60+RAL versus none) (student's t test). **C.** RAL induces *in vitro* mouse keratinocyte proliferation which is inhibited by anti-erbB1 and TIMP-3. Keratinocytes from SKH1 and DBA/1 mice were cultured in 96-well plates. On day 2 of culture, RAL (2 µM), monoclonal anti-human amphiregulin neutralizing antibody (100 ng/ml), monoclonal anti-human erbB1 neutralizing antibody (isotype IgG1) (100 ng/ml) or mouse recombinant TIMP-3 (100 ng/ml) was added to the cultures. Mouse IgG1 was used as a control of anti-erbB1. 48 hrs later 1 µCi of [^3^H]thymidine was added to each well. All experiments were done in triplicate and repeated 5 times. The results are presented as the mean incorporated counts per minute ± SEM of three wells per group. ***p<0.001(RAL versus none; anti-AR+RAL versus none; anti-erbB1+RAL versus none; TIMP3+RAL versus none; mouse IgG1 versus RAL) (student's t test). **D.** RAL and HAFi show a synergy *in vitro* which is CD44- and HB-EGF-dependent. Keratinocytes from SKH1, DBA/1 and CD44-/- mice were cultured in 96-well plates. On day 2 of culture, RAL (2 µM), HAFi (100 µg/ml), human HB-EGF (50 ng/ml) or mouse anti-human HB-EGF neutralizing antibody (100 ng/ml) was added to the cultures. 48 hrs later 1 µCi of [^3^H]thymidine was added to each well. All experiments were done in triplicate and repeated 5 times. The results are presented as the mean incorporated counts per minute ± SEM of three wells per group. ***p<0.001 (HAFi, RAL+HAFi and HB-EGF versus none; anti-HB-EGF+HAFi versus HAFi; anti-HB-EGF+RAL+HAFi versus RAL+HAFi); **p<0.01 (RAL versus none; HB-EGF versus none; anti-HB-EGF+RAL versus RAL; antiHB-EGF+HAFi versus HAFi) (student's t test).

To determine the effect of different retinoids on in vitro keratinocyte proliferation, we established primary cultures of keratinocytes from DBA/1 mice. Treatment of mouse keratinocytes with 2 µM of RAL resulted in slight but the most significant increase in keratinocyte proliferation when compared to retinoic acid (RA), retinol (ROL) or retinoyl palmitate (ROLP) (Figure 1A).

### RAL-induced HA synthesis is required for keratinocyte proliferation

To explore the role of HA synthesized by keratinocytes on RAL-induced keratinocyte proliferation, the keratinocytes of DBA/1 mice were treated with an HA synthesis inhibitor, 4-methylumbelliferone (4-MU), or hyaluronidase. RAL-induced keratinocyte proliferation was inhibited by 4-MU added to the cultures before or in the mean time with RAL (Figure 1B).

### *In vitro* proliferation of CD44-deficient keratinocytes is defective in response to RAL

To further address the importance of the presence of CD44 in this proliferative effect, we tested the ability of proliferation of keratinocytes of CD44-deficient (CD44^−/^) mice in response to RAL. In contrast to normal keratinocytes, no proliferation was observed in CD44^−/−^ cells (Figure 1C).

### Anti-HB-EGF, anti-erbB1 and TIMP-3 inhibits RAL-induced keratinocyte proliferation *in vitro*

Since HB-EGF is thought to be involved in retinoid-induced epidermal hyperplasia and because keratinocytes normally express several CD44 isoforms, including exon v3-containing variants (CD44v3) [18], which are substituted in heparan sulfate side chains and form a complex with matrix metalloproteinase 7 (MMP-7), heparin binding epidermal growth factor (HB-EGF) precursor (pro-HB-EGF) and its receptors (erbB4, erbB1) [14], [15], we first compared the ability of CD44^−/−^ and normal keratinocytes to proliferate in response to HB-EGF. HB-EGF (50 ng/ml) stimulated the proliferation of wild-type DBA/1 and, to a lesser extent, SKH1 hairless keratinocytes, whereas only a slight proliferation increase was observed in CD44-deficient keratinocytes (Figure 1C). We further analyzed the requirement of HB-EGF and its receptors in RAL-induced keratinocyte proliferation. Since erbB4 is not expressed in neither mouse [19] nor human [20] keratinocytes and erbB1 (epidermal growth factor receptor [EGFR]) is the principal receptor of HB-EGF in human keratinocytes [15], [20], we tested the cultured keratinocytes of SKH1 hairless and DBA/1 mice for response to RAL in the presence of anti-HB-EGF (100 ng/ml) or anti-erbB1 (100 ng/ml) neutralizing antibodies. Anti-HB-EGF significantly inhibited RAL-induced proliferative response in normal keratinocytes (Figure 1C). Blocking antibodies against amphiregulin (AR), a growth factor which fails to bind CD44v3 [21], did not inhibit keratinocyte proliferation (Figure 1D). In contrast, anti-erbB1 significantly blocked the proliferation of keratinocytes (Figure 1D). Since MMP-7 was shown to form a complex with its substrate pro-HB-EGF and CD44v3 on the surface of some cells and MMPs [14] and the related a disintegrin and metalloproteinase (ADAM) family proteases [22] activate HB-EGF, we also tested the effect of tissue inhibitor of metalloproteinase-3 (TIMP-3) which inhibits the effect of MMPs including MMP-7 and of ADAMs, on the keratinocyte proliferation induced by RAL. Incubation of the keratinocytes of SKH1 hairless and DBA/1 mice with TIMP-3 (100 ng/ml) abrogated the RAL-induced keratinocyte proliferation (Figure 1D).

### RAL and HAFi show a synergy in *in vitro* proliferative response of mouse keratinocytes which is CD44- and HB-EGF-dependent

To address the effect of the combination of different retinoids and HAFi on keratinocyte proliferation, we tested the response to 100 µg/ml of HAFi and 2 µM of ROL, RAL, RA and ROLP of cultured keratinocytes from the back skin of DBA/1 mice. Exposure to HAFi and RAL resulted in a more significant increase in keratinocyte proliferation than HAFi with other retinoids (Figure 1A). HAFi and RAL combination was more efficient in the proliferation of keratinocytes from the back skin of SKH1 hairless and DBA/1 mice than RAL or HAFi alone, whereas no proliferation was observed in CD44^−/−^ cells (Figure 1D). The proliferative response was slightly higher in DBA/1 keratinocytes (Figure 1C). Addition of blocking anti-HB-EGF antibodies (100 ng/ml) to RAL- and HAFi-treated cultures abrogated the proliferative response as in only RAL- or HAFi-treated cultures (Figure 1D).

### The effect of RAL and HAFi *in vivo* is CD44-dependent

**Figure 2:**
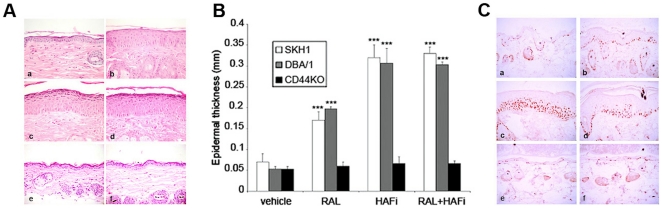
The effect of RAL and HAFi *in vivo*. **A.** The effect of RAL and HAFi *in vivo* on mouse skin. Histological sections of vehicle- (a), RAL- (b), HAFi- (c) or RAL- and HAFi- (d) treated DBA/1 and vehicle (e) or RAL- (f) treated CD44-/- mouse dorsal skin. Note the significant epidermal hyperplasia in DBA/1 but not in CD44-/- mice. **B.** Epidermal thickness in SKH1, DBA/1 and CD44-/- mouse dorsal skin measured with an ocular micrometer. Ten measurements were performed per mouse and the average value was calculated. The results are presented as the mean epidermal thickness ± SEM of six animals per group. ***p<0.001 versus vehicle (student's t test). **C.** Ki67 staining of vehicle- (a), RAL- (b), HAFi- (c) or RAL- and HAFi- (d) treated DBA/1 and vehicle (e) or RAL- (f) treated CD44-/- mouse dorsal skin.

Topical application of the combination of 0.05% RAL and 0.2% HAFi for 3 days resulted in a significant epidermal hyperplasia in the follicular (data not shown) and interfollicular epidermis in SKH1 hairless and DBA/1 mice (Figure 2A). This hyperplasia was more significant when compared to that induced by RAL alone, but it was comparable to that induced by HAFi alone (Figure 2B). In addition, topically applied RAL and HAFi significantly increased the number of Ki-67-positive cells in the epidermis and dermis (Figure 2C). The increase in epidermal Ki-67-positive cells was comparable to that with RAL or HAFi alone (data not shown). However, the increase in dermal Ki-67-positive cells was superior to that with RAL alone but it was not different when compared with the one with HAFi alone (data not shown). In contrast, topical application of RAL and HAFi, alone or in combination, to the back skin of CD44^−/−^ mice did not result in epidermal hyperplasia or keratinocyte proliferation (Figure 2A, B, C, data not shown). Topically applied RAL and HAFi, as HAFi alone but not RAL alone, led to an increased cellularity in the superficial and reticular dermis in SKH1 hairless and DBA/1 but not in CD44^−/−^ mice (data not shown).

### *In vivo* proliferative response of mouse epidermis to RAL is CD44-dependent

The effect of skin CD44 expression loss on epidermal hyperplasia induced by topically-applied RAL was addressed by comparing the histology of retinoid-treated skin areas of CD44^−/−^ and normal littermates. Topical application of 0.05% RAL for 3 days resulted in a significant epidermal hyperplasia and keratinocyte proliferation as determined by quantitation of Ki67 in the back skin of SKH1 hairless and DBA/1 mice, whereas no epidermal hyperplasia and keratinocyte proliferation was observed in CD44^−/−^ mice. (Figure 2A, B, C).

### RAL and HAFi have a synergistic effect on the protein and mRNA expression of CD44 in mouse skin

**Figure 3:**
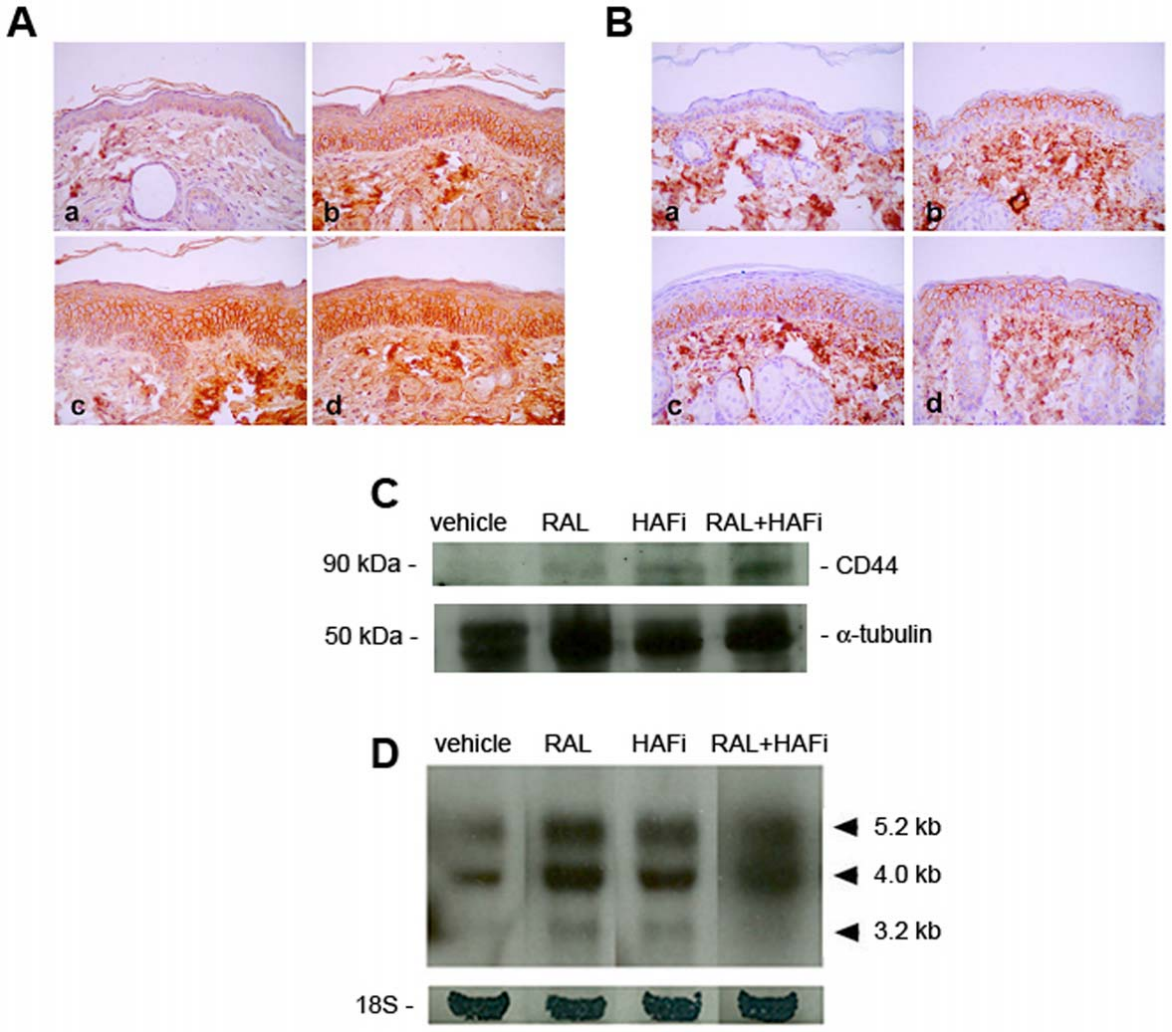
RAL and HAFi have a synergistic effect on the protein and mRNA expression of CD44 in mouse skin. **A.** Immunostaining of sections of vehicle- (a), RAL- (b), HAFi- (c) or RAL- and HAFi- (d) treated DBA/1 mouse dorsal skin with anti-CD44 antibodies. Note the hyperplasia and increase in diffuse CD44 expression in the epidermis in RAL-, HAFi- and RAL- and HAFi-treated mice. **B.** Immunostaining of sections of vehicle- (a), RAL- (b), HAFi- (c) or RAL- and HAFi- (d) treated DBA/1 mouse dorsal skin with anti-CD44v3 antibodies. Note the hyperplasia and increase in suprabasal CD44v3 expression in the epidermis in RAL-, HAFi- and RAL- and HAFi-treated mice. **C.** Western blot analysis for CD44 (∼90 kDa) performed on the epidermal proteins extracted from the RAL-, HAFi- and RAL- and HAFi-treated skin of SKH1 hairless mice. α-tubulin (50 kDa) has been used as a loading control. **D.** Northern blot analysis of CD44 RNA expression in RAL-, HAFi- and RAL- and HAFi-treated skin of DBA/1 mice. Note the increase of the three CD44 transcripts (5.2, 4.0 and 3.2 kb) in RAL-, HAFi- and RAL- and HAFi-treated skin.

Immunostaining of vehicle-treated back skin of DBA/1 mice revealed the standard expression of CD44 in basal and suprabasal keratinocytes (Figure 3A, a). However, topical application of RAL and HAFi for 3 days significantly increased CD44 expression in follicular (data not shown) and interfollicular keratinocytes (Figure 3A, d), as shown by using an antibody recognizing all CD44 isoforms. The staining intensity for CD44 was stronger in HAFi alone- and RAL- and HAFi-treated epidermis than the one treated with RAL alone (Figure 3A, b, c, d). An antibody which specifically recognizes CD44v3 isoform revealed increased expression of CD44v3 in suprabasal keratinocytes with the exception of the basal and granular layer after topical application of RAL and HAFi (Figure 3B, a, b, c, d). The staining for CD44v3 was stronger in the upper suprabasal layers in the RAL alone- and RAL- and HAFi-treated epidermis when compared to the one treated with HAFi alone (Figure 3B, b, c, d). Although RAL seemed to increase the expression of CD44 more at the RNA level (Figure 3D), the protein expression of CD44 was more augmented by HAFi (Figure 3C). CD44 expression in RAL- and HAFi-treated SKH1 hairless or DBA/1 mouse back skin was more significantly increased than in RAL alone- or HAFi alone- treated skin of these mice both at the RNA and protein levels (Figure 3C, D).

### RAL and HAFi have a synergistic effect on the epidermal and dermal HA production in mouse skin

**Figure 4:**
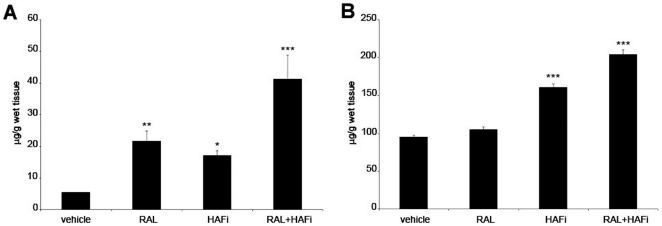
RAL and HAFi have a synergistic effect on the epidermal and dermal HA production in mouse skin. Epidermal (A) and dermal (B) HA content of vehicle-, RAL, HAFi- or RAL- and HAFi-treated skin of SKH1 hairless mice quantified by an enzyme-linked binding protein assay. The results are presented as the mean HA concentration ± SEM of six animals per group. ***p<0.001 versus vehicle; **p<0.01 versus vehicle; *p<0.05 versus vehicle (student's t test).

Quantification of epidermal and dermal HA was performed by an enzyme-linked binding protein assay on the RAL-, HAFi, and RAL and HAFi-treated epidermis and dermis of SKH1 hairless (not shown) and DBA/1 mice. RAL alone seemed to increase more epidermal HA than HAFi alone (Figure 4A). In contrast, HAFi alone resulted in more HA in dermis than RAL alone (Figure 4B). RAL and HAFi combination more significantly increased epidermal and dermal HA than with RAL or HAFi alone (Figure 4A, B).

### RAL and HAFi have a synergistic effect on the protein expression of pro-HB-EGF in mouse skin

**Figure 5:**
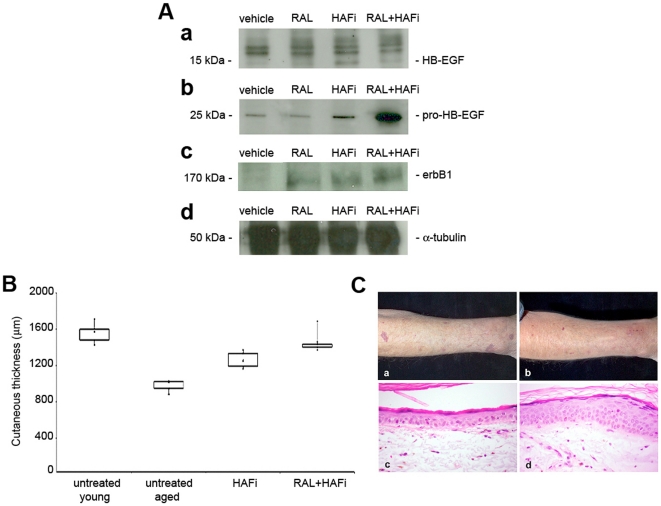
The synergistic effect of RAL and HAFi in the protein expression of pro-HB-EGF in mouse skin and in human skin atrophy. **A.** Western blot analysis on the protein extracts of vehicle-, RAL-, HAFi- or RAL- and HAFi-treated SKH1 hairless mice for active HB-EGF (∼15 kDa) (a), pro-HB-EGF (∼25 kDa) (b) and erbB1 (∼170 kDa) (c) and loading control α-tubulin (50 kDa) (d). **B.** RAL and HAFi show synergy in the correction of dermatoporosis. Skin thickness in untreated young, untreated atrophic aged, HAFi alone-treated atrophic aged or RAL and HAFi-treated atrophic aged human skin measured by echography. The results are presented as box-plots with median value (▴). p = 0.001 (young untreated versus atrophic aged untreated); p<0.01 (atrophic aged untreated versus atrophic aged treated with HAFi); p<0.001 (atrophic aged untreated versus atrophic aged treated with RAL and HAFi); p<0.05 (atrophic aged treated with HAFi versus atrophic aged treated with RAL and HAFi) (nonparametric Mann-Whitney U test). **C.** Clinical view of atrophic human forearm skin before (a) and 1 month after topical treatment with RAL and HAFi (b). Note the decrease of wrinkles, hematoma and pseudoscars, and smoothening of the skin after RAL and HAFi treatment. Histological view of atrophic human forearm skin before (c) and 1 month after topical treatment with RAL and HAFi (d). Note the significant epidermal hyperplasia, decrease of elastosis and increase of collagen in the dermis after RAL and HAFi treatment.

To address the effect of the combination of topically-applied RAL and HAFi on the expression of CD44v3, pro- and active HB-EGF and its receptor erbB1 in vivo, we performed a western blot analysis on the protein extracts of RAL- and HAFi-treated SKH1 hairless mice. RAL alone-treated epidermis showed a slight increase of CD44v3 (data not shown), active HB-EGF and erbB1 protein expression when compared to vehicle-treated epidermis (Figure 5A, a, c). Protein content of pro-HB-EGF was not increased by RAL (Figure 5A, b). HAFi alone-treatment resulted in an increase of CD44v3 (data not shown), pro- and active HB-EGF, and erbB1 protein expression when compared to vehicle-treated epidermis (Figure 5A, a, b, c). HAFi increased active HB-EGF more than RAL (Figure 5A, a). There was no difference between the RAL and HAFi in terms of the increase of erbB1 (Figure 5A, c). There was no difference in erbB3 expression between RAL- and vehicle-treated epidermis (data not shown). No expression of erbB2 or erbB4 was detected in mouse skin (data not shown). RAL did not influence the expression of MMP-7 (data not shown). RAL and HAFi-treatment showed a synergy only on the expression of pro-HB-EGF (Figure 5A, b).

### RAL and HAFi show synergy in the correction of human skin atrophy

Topical application of RAL and HAFi to the atrophic forearm skin of elderly dermatoporosis patients for 1 month not only corrected the skin atrophy but also caused marked skin hyperplasia. This effect was more significant than RAL alone (data not shown) or HAFi alone (Figure 5B). Clinically, the wrinkles, hematoma and pseudoscars seen in atrophic skin decreased and smoothening of the skin was observed after RAL and HAFi treatment (Figure 5C, a, b). Histologically, the epidermal hyperplasia observed after RAL and HAFi treatment was associated with a decrease of elastosis an increase of collagen in the dermis (Figure 5C, c, d).

## Materials and Methods

### Topical retinoid preparation

RAL was used at the concentration of 0.05% compounded in an oil-in-water cream [11].

### Preparation of HAFi

HA from rooster comb (IAL) for clinical application was provided by Transbussan (Geneva, Switzerland). HAFi: Fragmentation products of HA of medium size (50’000–400’000 Da) was prepared using a Braun sonifier for 30 min at 400 W on ice. The fragments were then separated on a Sephacryl S-400 HR 4x80 cm size exclusion gel filtration column (Pharmacia Diagnostics, Uppsala, Sweden). The column was calibrated using Pullulan standards (Showa-Denko, Kawasaki, Japan) from 20’000 to 800’000 Da. 100 fractions of 10 ml each were collected from the column with a Frac. 100 fraction collector (Pharmacia) at a flow rate of 1 ml/min. The HA concentration of each sample was determined by a colorimetric dosage according to a BCA reducing sugar assay and an elution profile for the fragments was obtained. The fractions corresponding to the 50’000–400’000 Da were pooled, dialyzed against distilled water and lyophilized. The fragments were separated by 15% polyacrylamide gel (TBS) electrophoresis and visualized with an alcian blue and silver staining. HAFi were prepared in the form of cream in a neutral vehicle.

### Treatment of mice

Groups of five adult (>3 month-old) SKH1 hairless, DBA/1 (The Jackson Laboratory, Bar Harbor, ME) or CD44-deficient (CD44^−/−^) mice [14] were used. HAF, RAL or vehicle cream samples of 0.5 g were applied twice daily for 3 days to the dorsal skin of SKH1 hairless, DBA/1 or CD44^−/−^ mice. The daily amount of RAL delivered corresponded to 250 µg. Animals were sacrificed two hours after the last application. All animals were handled in strict accordance with good animal practice as defined by the relevant national and/or local animal welfare bodies, and all animal work was approved by the Ethical Commission on Animal Experimentation of the University of Geneva and the Cantonal Veterinary Office of Geneva (ethics approval code 31.1.1035/2368/I).

### Treatment of healthy subjects and patients

7 healthy young adults (7 M) between 19 and 32 years (mean age 25.5 years), 3 patients with advanced age-related dermatoporosis (2 F; 1 M) between 60 and 88 years (mean age: 76 years) and 3 patients with dermatoporosis due to prolonged use of oral corticosteroids (3 F) between 74 and 86 years (mean age: 81 years) (for HAFi only treatment), and 3 patients with advanced age-related dermatoporosis (2 F; 1 M) between 75 and 92 years (mean age: 84 years) and 3 patients with dermatoporosis due to prolonged use of oral corticosteroids (2 F; 1 M) between 63 and 77 years (mean age: 71 years) (for RAL and HAFi treatment) were included in this study after obtaining written informed consent. Clinical studies were conducted with the authorization and according to the guidelines of Ethical Commission on Human Research of the University Hospital of Geneva. 0.05% RAL, 1% HAFi or vehicle cream samples of 0.5 g were applied twice daily for 3 months on the posterior side of the right or left arm, respectively.

### Histology

Dorsal skin samples were fixed in 10% phosphate-buffered formaldehyde, embedded in paraffin, and processed for histological analysis. Sections were cut at 5 µm, mounted onto slides, and stained with hematoxylin-eosin according to standard procedures.

### Staining of skin sections for CD44, CD44v3, HABP, Ki-67

Paraffin-embedded sections (5 µm) were mounted onto slides, de-waxed in xylene, rehydrated in a graded ethanol series and prepared for immunoperoxidase staining according to standard procedures. Primary antibodies included: anti-CD44v3 (Bender MedSytems, Vienna, Austria; 1∶100), anti-Ki-67 (Dako, Glostrup, Denmark; 1∶20). After staining with the primary reagent for 1 hr at room temperature, sections were washed, incubated with biotinylated affinity-purified secondary antibody or with biotinylated anti-CD44 (PharMingen, San Diego, CA; 2.5 µg/ml) for 30 min at room temperature, washed, and treated with avidin-biotin-peroxidase for 30 min at room temperature. The sections were then washed with buffer and incubated in 0.05% DAB (3,3′-diaminobenzidine; Sigma) and 0.03% H_2_O_2_ in phosphate buffer at room temperature. All sections were examined under a Zeiss axiophot microscope using appropriate filters.

### Epidermal and cutaneous thickness measurements

Epidermal thickness of mice was measured by a graded ocular and multiplied by 10 to correct the scale. Cutaneous thickness measurements of the healthy subjects and patients were performed using a skin ultrasound system (Episcan, Longport Inc., Glen Mills, PA).

### Cell proliferation

Epidermal and dermal Ki-67 positive cells were counted in ten fields per section at 40× magnification and the average value was calculated.

### RNA isolation and Northern blotting

Total RNA from the back skin of SKH1 hairless, DBA/1 and CD44*^−/−^* mice topically treated with RAL was prepared using a TRIzol reagent kit (Invitrogen) and digested with RNase-free DNase 1 (Ambion). Eight micrograms of isolated RNAs were separated electrophoretically on a 1% agarose gel containing glyoxal and transferred onto a nitrocellulose membrane. The membrane was UV-cross-linked and probed with [^32^P]UTP-labeled hyaluronate synthase (HAS) cDNA (1×10^6^ cpm/ml). Hybridization with CD44 probe prepared using random primers and CD44 fragment purified from *Hind*III-*Eco*RI-digested mCD44H-pBS plasmid template (Kaya *et al*, 1997) was performed overnight at 60°C in 50% formamide, 1× sodium chloride-sodium citrate, 5× Denhardt's reagent and 0.2% tRNA. The membrane was washed twice at 68°C for 30 minutes in 0.1× sodium chloride-sodium citrate and 0.1% sodium dodecyl sulfate. Kodak (Rochester, NY) X-Omat AR film was exposed overnight at −70°C. The integrity of total RNA was good, and the ratio of 28S and 18S ribosomal RNAs was ∼2∶1 in all samples. The hybridization signals were quantitated by scanning the autoradiograms with a laser densitometer and using the ImageQuant software.

### Western blot analysis

Frozen mouse epidermis or dermis and human biopsy samples were incubated in extraction buffer containing 20 mM Tris/HCl (pH 7.5), 100 mM NaCl, 10 mM EDTA, 1% SDS, 10% glycerol and protease inhibitor cocktail (complete™ Boehringer, Biberach, Germany), minced, polytron-homogenized and sonicated on ice. Cell culture extracts were treated with the same buffer. Homogenates were spun in a microfuge for 20 min, and the soluble fraction was extracted and subjected to western blot analysis with appropriate antibodies.

Samples were loaded in nonreducing SDS sample buffer, subjected to electrophoresis, and transblotted onto 0.45 µm pore-size nitrocellulose membrane. Antibodies used for Western blot analysis were anti-CD44 standard (Bender MedSytems); anti-CD44v3 (Bender MedSystems); anti-MMP-7 (G-20) (Santa Cruz Biotechnology); anti-pro-HB-EGF (M-18) (Santa Cruz Biotechnology); anti-HB-EGF neutralizing antibody (R&D Systems, Minneapolis, MN); and anti-erbB1 and anti-erbB4 (Santa Cruz Biotechnology).

### *In vitro* keratinocyte proliferation assay

Epidermal keratinocytes were isolated and cultured in 96-well plates (Becton Dickinson) as described previously [16]. On day 2 of culture, HAFi (100 µg/ml), RAL (2 µM), RA (2 µM), ROL (2 µM), ROLP (2 µM), 4-MU (0.6 mM) (Sigma), hyaluronidase (1 U/ml) (Sigma), RAL+HAFi, RAL+HAFi, RA+HAFi, ROL+HAFi or ROLP+HAFi, monoclonal anti-human amphiregulin (AR) neutralizing antibody (100 ng/ml), monoclonal anti-human erbB1 neutralizing antibody (isotype IgG1) (100 ng/ml), mouse recombinant TIMP-3 (100 ng/ml) or human HB-EGF (5 ng/ml) (R&D Systems), TPA (1 ng/ml) (Sigma), human EGF (50 ng/ml) (Sigma), in the presence or absence of anti-HB-EGF neutralizing antibody (10 ng/ml) (R&D Systems), was added to the cultures. Mouse IgG1 was used as a control of anti-erbB1. 48 hrs later 1 µCi of [^3^H]thymidine (Amersham, Buckinghamshire, UK) was added to each well. Isotope incorporation was evaluated 24 hrs later in a Beckman LS 1801 β counter (Beckman Coulter, Fullerton, California). All experiments were done in triplicate and repeated 5 times, and the results were expressed as the mean of incorporated counts per minute for each condition tested.

### Determination of HA synthesis *in vitro*

Keratinocytes were cultured in six-well culture plates (Falcon, Becton Dickinson and Co.) as described above. On day 12 of culture, HAFi (100 µg/ml), a RAL (2 µM) or RAL+HAFi were added to the cultures. 48 hrs later HA synthesis was determined by the quantification of HA in the culture supernatants by an enzyme-linked binding protein assay as described above.

## Discussion

In this study we provide evidence that RAL induces an in vitro and in vivo proliferative response of keratinocytes which is mediated by a CD44-dependent pathway and that RAL and HAFi show a synergy in vitro and in vivo with a potential therapeutic effect in dermatoporosis.

In our previous study we have shown for the first time that HAFi induce an in vitro keratinocyte proliferation [15]. This proliferative response was inhibited, like for RAL, by anti-erbB1 blocking antibodies and TIMP-3, an inhibitor of MMPs and ADAMs, suggesting that the RAL- or HAFi- induced in vitro proliferative response requires erbB1 and MMPs/ADAMs. In the present study we show that RAL and HAFi have a synergistic effect on the in vitro proliferation of keratinocytes. Our previous and present observations also provide evidence that CD44 and HB-EGF are required for RAL- or HAF-mediated in vitro keratinocyte proliferation. The absence of a proliferative response of CD44^−/−^ keratinocytes to HB-EGF is consistent with the notion that HB-EGF interaction with its receptors requires presentation by heparan sulfate side chains of CD44v3-containing isoforms which appear to constitute a major fraction of heparan sulfate proteoglycans expressed on the surface of normal keratinocytes [16].

Our observations show that topical application of RAL and HAFi to normal mouse skin leads to: (1) significant epidermal hyperplasia accompanied by increased cell proliferation in epidermis and dermis, (2) increase of CD44 and its CD44v3 isoform, pro- and active HB-EGF, and erbB1 expression, (3) increase of epidermal and dermal HA.

Significant epidermal hyperplasia and the increased cell proliferation in epidermis and dermis observed in SKH1 hairless and DBA/1 mice with topical RAL and HAFi was superior to the one induced by RAL alone but comparable to the one resulted from HAFi alone. The comparable result between HAFi alone and RAL and HAFi combination is most likely due to the saturation of proliferative capacity of keratinocytes.

Topical RAL and HAF increase the expression of CD44 at the RNA and protein levels in mouse skin. This increase is more significant then the one after RAL or HAFi alone, indicating the synergistic effect of these two molecules on CD44 expression. RAL and HAF also upregulate the CD44v3 isoform in the epidermis. However, no synergy was observed in CD44v3 expression. The synergistic effect of RAL and HAFi on keratinocyte CD44 expression might be due either to a combined effect of RAL and HAFi on CD44 gene transcription or to a presently unknown feedback mechanism in order to upregulate the cell surface CD44 for internalization and degradation of increased HA in interkeratinocyte spaces.

Topical application of RAL and HAF resulted in a significant increase of pro-HB-EGF and HB-EGF protein levels in mouse epidermis as well as a slight increase in erbB1 protein expression. Although the increase in the protein levels of active HB-EGF was not more significant than HAFi alone, a clear synergy was observed in the induction of pro-HB-EGF expression. The production of more HB-EGF after RAL and HAFi application might prolong the proliferative effect on the epidermis by the generation of more HB-EGF over time which interacts with its receptor erbB1 which is also upregulated. In our previous study we showed the association of CD44 with erbB1 in keratinocytes *in vitro* and *in vivo* [15]. This observation is consistent with the notion that topical HAFi upregulates the molecules shown to form a complex with CD44 in mouse uterine and mammary epithelia [14], and that erbB1 blocking antibodies have the same abrogating effect as the absence of CD44 on *in vitro* keratinocyte proliferation. It seems that erbB4 which serves as the receptor for HB-EGF in uterine and mammary epithelia is replaced by erbB1 in keratinocytes, and that the same or similar CD44 complex is most likely present on keratinocytes as well [15].

**Figure 6:**
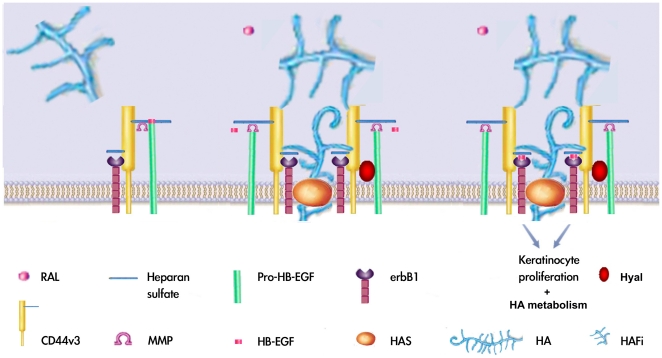
Hypothetical assembly of CD44v3/HB-EGF/erbB1/HAS/Hyal (hyalurosome) complex and the mechanism of RAL and HAFi-induced keratinocyte proliferation. Schematic representation of the assembly of the putative complex: HAFi mediates CD44v3 and heparan-sulfate-bound pro-HB-EGF aggregation, resulting in the recruitment and activation of an MMP/ADAM. RAL increases the endogenous HA production by induction of HAS which will contribute to the aggregation of CD44v3 and pro-HB-EGF and to the activation of MMP/ADAM. Pro-HB-EGF is cleaved by the MMP/ADAM and the resulting active moiety binds and activates erbB1, generating proliferation signals. Endogenous HA synthesis is controlled by HA-degrading enzyme hyaluronidase (Hyal).

Topically applied RAL and HAF also show a synergy in the increase of the epidermal and dermal HA content in mouse skin. RAL alone increases more HA in epidermis than HAFi alone; in contrast, HAFi alone results in more dermal HA than RAL alone. RAL and HAFi application more significantly augments HA in epidermis and dermis than with RAL or HAFi alone. We recently showed that the increase in cutaneous HA by RAL is due to the increased expression of HA-polymerizing enzymes, hyaluronate synthase (HAS)s 1, 2 and 3 [7]. The synergistic effect on epidermal and dermal HA increase is most likely due to the upregulation of HAS1, HAS2 and HAS3 also by HAFi [15]. In our previous study we demonstrated that topically applied HAFi traverse the skin, which also contributes the dermal increase of HA [15]. Exogenous RAL and HAFi as well as the endogenous HA might play an important role in the activation of CD44 platform which we call hyalurosome complex which regulates keratinocyte proliferation and HA metabolism (Figure 6).

Recently we have shown that topical application of HAFi restores atrophic skin lesions in humans by a CD44-dependent mechanism [15]. Topical RAL and HAFi application resulted in skin hyperplasia in dermatoporotic human skin which was more significant than the one with RAL alone and HAFi alone. This synergistic effect was observed as early as 1 month after initiation of treatment and was accompanied by a significant clinical improvement.

Taken together, our observations suggest that RAL-induced in vitro and in vivo proliferative response of keratinocytes is mediated via a CD44-dependent pathway and requires the presence of HB-EGF, erbB1 and an MMP or ADAM. Therefore CD44-dependent recruitment and processing of HB-EGF and EGF receptors may provide a mechanism for cell growth modulation induced by topical retinoids. Our results also indicate that RAL and HAFi combination may be used to develop novel therapeutic strategies for skin atrophy.
